# Phytochemical Analysis, Antioxidant, Analgesic, Anti-Inflammatory, Hemagglutinin and Hemolytic Activities of Chemically Characterized Extracts from *Origanum grosii* (L.) and *Thymus pallidus* (L.)

**DOI:** 10.3390/plants13030385

**Published:** 2024-01-28

**Authors:** Hind Zejli, Amira Metouekel, Otmane Zouirech, Imane Maliki, Abdelfattah El Moussaoui, Aziza Lfitat, Fatima Zahra Bousseraf, Khalid S. Almaary, Hiba-Allah Nafidi, Farid Khallouki, Mohammed Bourhia, Mustapha Taleb, Abdelfattah Abdellaoui

**Affiliations:** 1 Laboratory of Engineering, Electrochemistry, Modeling and Environment, Faculty of Sciences Dhar El Mahraz, Sidi Mohamed Ben Abdellah University, Fez 30000, Morocco; hind.zejli@usmba.ac.ma (H.Z.);; 2 Euromed Research Center, Euromed Faculty of Pharmacy, Euromed University of Fes (UEMF), Meknes Road, Fez 30000, Morocco; 3 Laboratories of Natural Substances, Pharmacology, Environment, Modeling, Health, and Quality of Life, Faculty of Sciences Dhar El Mahraz, Sidi Mohamed Ben Abdellah University, Fez 30000, Morocco; 4 Laboratory of Health and Environment, Department of Biology, Moulay Ismail University, Meknes 50050, Morocco; imanemaliki16@gmail.com; 5 Plant Biotechnology Team, Faculty of Sciences, Abdelmalek Essaadi University, Tetouan 93002, Morocco; 6 Department of Botany and Microbiology, College of Science, King Saud University, P.O. Box 2455, Riyadh 11451, Saudi Arabia; 7 Department of Food Science, Faculty of Agricultural and Food Sciences, Laval University, Quebec City, QC G1V 0A6, Canada; 8 Team of Ethnopharmacology and Pharmacognosy, Biology Department, Faculty of Sciences and Techniques, University Moulay Ismail, Errachidia 52000, Morocco; 9 Department of Chemistry and Biochemistry, Faculty of Medicine and Pharmacy, Ibn Zohr University, Laayoune 70000, Morocco

**Keywords:** oregano, thyme, HPLC, anti-inflammatory, analgesic, DPPH, hemolytic

## Abstract

*Origanum grosii* (L.) and *Thymus pallidus* (L.) are medicinal plants recognized for their uses in traditional medicine. In this context, the aim of this article is to highlight the results of a phytochemical analysis (HPLC), with particular emphasis on the antioxidant (DPPH, TAC, and FRAP), analgesic, anti-inflammatory, haemagglutinin-test-related, and hemolytic activities of the total extracts of these plants. Phytochemical analysis via HPLC revealed that licoflavone C (30%) is the main compound in *Origanum grosii*, while hesperidin (43%) is found in *T. pallidus*. Evaluation of the antioxidant capacity of *Origanum grosii* and *Thymus pallidus* using the DPPH, TAC, and FRAP methods revealed an IC50 of the order of 0.085 mg/mL and 0.146 mg/mL, an EC50 of the order of 0.167 mg/mL and 0.185 mg/mL, and a total antioxidant capacity of between 750 mg EQ/g and 900 mg EQ/g, respectively. Analgesic evaluations revealed writhes inhibition of the order of 97.83% for *O. grosii* and 90% for *T. pallidus*. In addition, both plant extracts showed limited hemolytic activity, not exceeding 30% at a concentration of 100 mg/mL. Evaluation of the anti-inflammatory potential showed edema inhibition of the order of 94% (800 mg/kg) for *O. grosii* and 86% (800 mg/kg) for *T. pallidus*. These results highlight the potential applications of these extracts in pharmacological research.

## 1. Introduction

With its diverse ecosystems and geographical features, Morocco is home to an exceptional variety of plant species, making it a treasure trove of botanical resources [1]. For centuries, traditional Moroccan medicine has relied on the use of plants to treat various health problems and improve general well-being [2]. Ethnobotanical studies carried out in Morocco have documented the wealth of indigenous knowledge of medicinal plants, which has been passed down from generation to generation [3].

Among the many plants used in traditional medicine, thyme (*Thymus pallidus*) and oregano (*Origanum grosii*) have emerged as two leading species due to their widespread application and perceived therapeutic benefits [4]. Thyme, known locally as “zaaitra”, is used for its antiseptic, anti-inflammatory, and expectorant properties [5]. Oregano, or “za’atar“, is valued for its antibacterial, antifungal, and antioxidant properties [6]. These plants are deeply rooted in Moroccan culture, not only as culinary herbs but also as remedies for ailments ranging from respiratory disorders to digestive problems [7].

In traditional medicine, plant use knowledge is often passed down orally from generation to generation. However, with cultural and lifestyle changes, this invaluable knowledge is at risk of being lost or forgotten [8]. This is why ethnobotanical surveys play a crucial role in documenting and preserving this intangible cultural heritage, providing valuable information on the therapeutic potential of medicinal plants [9].

The growing interest in traditional herbal remedies also highlights the importance of understanding the safety and efficacy of these treatments [10]. With thyme and oregano gaining importance in global health and wellness markets, it is becoming essential to explore their chemical compositions and potential bioactive constituents. High-performance liquid chromatography (HPLC) is a powerful tool for unraveling the complex phytochemical profiles of these plants, enabling the identification and quantification of various compounds that contribute to their medicinal properties [11,12].

The foundations of this research were laid by an ethnobotanical survey conducted within the Ribat El Kheir region. From the extensive list generated by the survey, we strategically selected two plants: thyme and oregano. Our primary approach revolved around a comprehensive analysis of the total extract, a preparation closely aligned with traditional usage as reported by the survey participants. This investigation encompassed an exploration of their antioxidant activity. Concurrently, we endeavored to identify the presence of lectins or other hemagglutination-inducing compounds, which could have a protective influence on red blood cells. Furthermore, we assessed the extracts’ hemolytic activity, offering initial insights into their potential toxicity.

After these evaluations, our focus shifted toward investigating the analgesic and anti-inflammatory properties of these total extracts in mouse models. Throughout our inquiry, a pivotal goal remained constant: to unravel the safety profile associated with the utilization of these time-honored medicinal plants. Our overarching objective is to ensure their responsible application, safeguarding the well-being of generations to come.

## 2. Result and Discussions

### 2.1. Phytochemical Study

**Figure 1:**
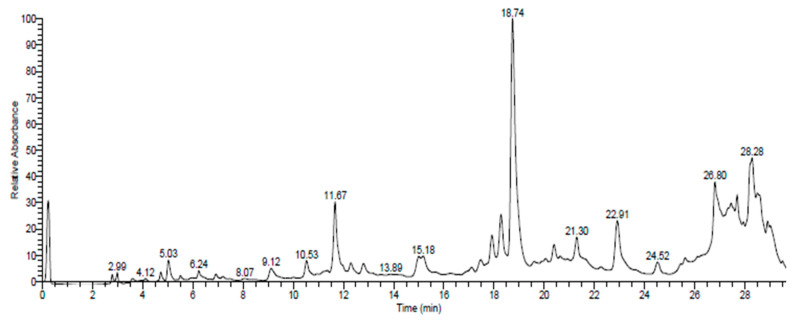
Chromatographic profile (UHPLC-DAD-ESI/MS) of P1 extracts at 280 nm.

**Figure 2:**
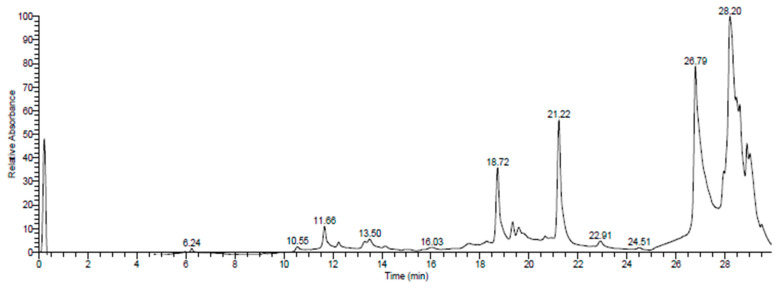
Chromatographic profile (UHPLC-DAD-ESI/MS) of P2 extracts at 280 nm.

The UHPLC-DAD-ESIMS analysis provided a comprehensive view of the chemical compositions of the total extracts from *O. grosii* and *T. pallidus* (Table 1, Figure 1 and Figure 2). These results carry particular significance when related to the therapeutic activities explored in this study. The identification of specific compounds, along with their respective retention times, sheds light on the potential bioactive molecules present in these extracts.

One prominent observation was the differing levels of hesperidin and rutin in the extracts. Hesperidin, detected in relatively high amounts in the *T. pallidus* extract, has a well-documented history of exhibiting potent antioxidant properties. Moreover, numerous studies have reported its efficacy in attenuating inflammatory responses [13]. These findings indicate that thyme may hold promise as an anti-inflammatory agent, particularly due to its high content of hesperidin. Conversely, the *O. grosii* extract is abundant in rutin, a compound renowned for its antioxidant and anti-inflammatory activities [14]. With a substantial presence of rutin in oregano, this extract is likely to exert robust antioxidant and anti-inflammatory effects. Furthermore, the presence of compounds like 4-*O*-Feruloylquinic acid and licoflavone C in these extracts’ hints at their potential contributions to the observed activities. The diverse compositions of oregano and thyme underscore the importance of harnessing these natural sources for therapeutic applications, further emphasizing the need for additional studies to elucidate their precise mechanisms and therapeutic potentials.

### 2.2. Antioxidant Capacity

The results from the DPPH and FRAP assays for *O. grosii* and *T. pallidus*, with BHT used as the standard for DPPH and quercetin as the standard for FRAP, highlight the antioxidant potential of these herbal extracts.

In comparison to the synthetic antioxidant BHT, *O. grosii* exhibited a DPPH value of 0.085 ± 0.004 mg/mL, suggesting its effectiveness in scavenging free radicals. This aligns with the results of previous studies by Jacob P et al. (2019), who reported similar DPPH values for oregano extracts, emphasizing their notable antioxidant activity [15].

*T. pallidus*, on the other hand, displayed a slightly higher DPPH value of 0.146 ± 0.02 mg/mL compared to BHT (Table 2). While it may have a slightly lower radical-scavenging ability, it still showcases significant antioxidant potential. This aligns with prior research in which thyme extracts were investigated and comparable DPPH values were noted, indicating their strong free-radical-scavenging capabilities [16,17,18].

When examining the FRAP values, *O. grosii* demonstrated an FRAP value of 0.167 ± 0.02 mg/mL (Table 2), indicating its robust reducing power in comparison to the quercetin standard. Researchers who conducted a study on oregano extracts found similar FRAP values, supporting the extract’s strong reducing capacity. *T. pallidus* exhibited a slightly higher FRAP value of 0.185 ± 0.04 mg/mL compared to quercetin, reinforcing its potential as a potent antioxidant with notable reducing power. These findings echo the work of Hossain et al. (2008), who investigated thyme and oregano plants and reported that oregano showed significant FRAP values, underlining oregano’s impressive reducing capabilities [19].

**Figure 3:**
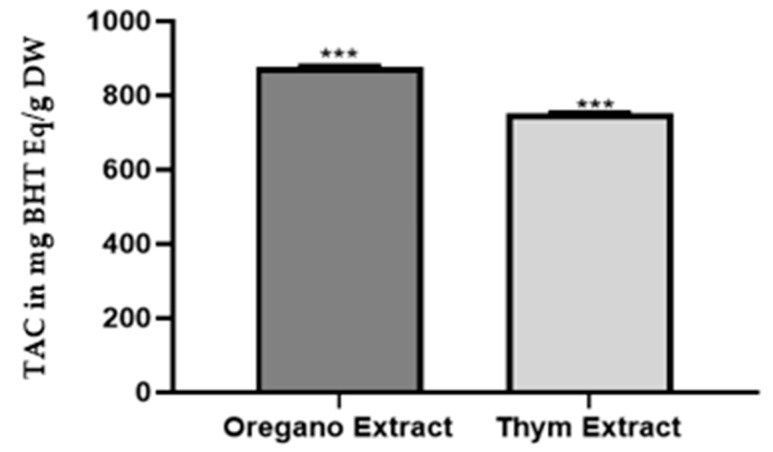
The total antioxidant capacity of *O. grosii and T. pallidus* extracts. Values marked with the same number of stars (***) in the chromatogram are not significantly different (*p* < 0.05).

The data provided in the histogram show the TAC results for three repetitions of oregano extract and thyme extract experiments, expressed in milligram equivalents of BHT per gram of dry weight (mg Eq of BHT/g DW). This result indicates that both plant extracts have antioxidant ability, but oregano has a slightly higher antioxidant capacity compared to thyme (Figure 3). These findings align with those from previous studies investigating the antioxidant potential of oregano and thyme extracts [20,21].

**Figure 4:**
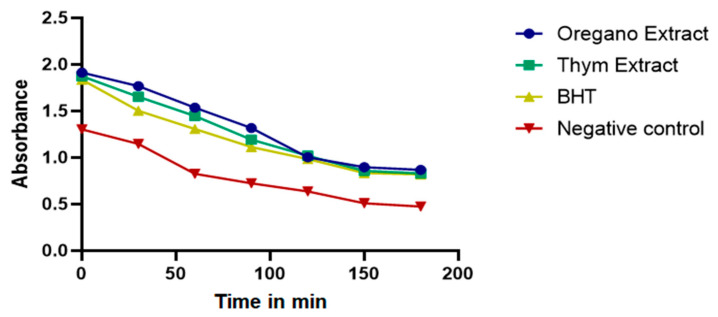
Absorbency of β-carotene at 470 nm in the presence of oregano EO, BHT, and negative control.

The graph below illustrates changes in absorbance over time for three substances (Figure 4), namely, oregano extract, thyme extract, and BHT, in the beta-carotene bleaching test; here, a decrease in absorbance signifies bleaching and is the primary indicator of antioxidant activity [22]. Importantly, the rate of decrease in absorbance compared to the positive control (BHT) provides valuable insights.

**Figure 5:**
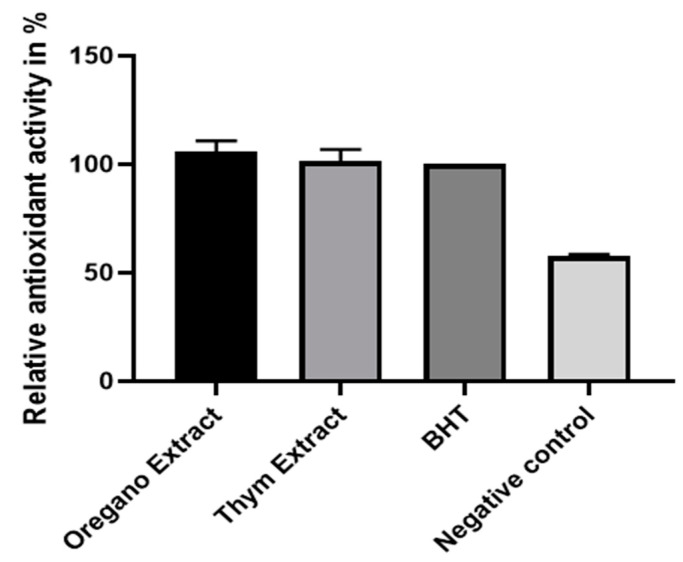
Relative antioxidant activity of *O. grosii* and *T. pallidus* extracts, BHT, and negative control.

As can be observed, it is evident that both oregano extract and thyme extract exhibited decreases in absorbance over time, indicating the bleaching of beta-carotene and, consequently, their antioxidant activity. However, how their absorbance decreases differs from that of the positive control (BHT); the noteworthy point is that the extracts from both plants display a slower rate of absorbance decrease compared to BHT (Figure 5). Our results align with those of previous studies investigating the antioxidant potential of natural extracts [23].

The histogram represents the relative percentages of antioxidant activity in the beta-carotene bleach test for oregano extract, thyme extract, BHT (butylated hydroxytoluene), and the negative control. The values were calculated relative to the synthetic positive control, BHT, and are presented as the mean of three replicates with standard deviation.

According to the averages, oregano extract shows the highest antioxidant activity among the samples, with an average relative antioxidant activity of around 108.9%, followed by thyme extract at around 100.9% and BHT at 100% (Figure 5). This suggests that oregano extract has the strongest antioxidant potential in this specific test, and the presence of phenolic compounds and flavonoids in oregano and thyme supports the antioxidant activities observed. These compounds are known for their ability to protect against oxidative stress and prevent the oxidation of sensitive molecules such as beta-carotene [24].

### 2.3. Hemagglutination

#### 2.3.1. Phyto-Hemagglutination Assay

Lectins were originally identified due to their capacity to induce erythrocyte agglutination, a technique that remains the easiest and most convenient approach for detecting lectin presence [25].

**Figure 6:**
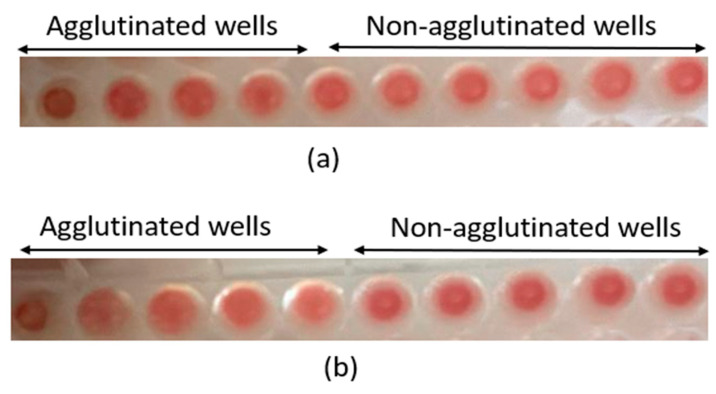
(**a**) Hemagglutination limit of *O. grosii,* for which observation with the naked eye was possible; (**b**) hemagglutination limit of *T. pallidus*, for which observation with the naked eye was possible.

Table 3 illustrates the agglutination of rat erythrocytes by the total extract of oregano and thyme. The hemagglutination activity of the lectins in the studied extracts is positive. A significant agglutination of erythrocytes was observed after 30 min of incubation. This agglutination was visible to the naked eye (Figure 6). This outcome indicates that the studied plants contain lectins. The interaction between lectins and red blood cells typically occurs when lectins are deposited into a well containing the erythrocytes. This interaction leads to the formation of a homogeneous gel-like mass, known as the hemagglutination phenomenon [26].

#### 2.3.2. Hemagglutination Limit Test

The hemagglutination limit test assesses the minimum concentration that induces hemagglutination activity. Hemagglutination activity is expressed as a titer, which is the reciprocal of the highest dilution ratio that shows hemagglutination.

The minimal concentrations causing erythrocyte hemagglutination are presented in Table 4 below: the minimum hemagglutination activity of the P1 extract is 1/20, while it is 1/50 for the P2 extract.

The results of this study highlight the significant hemagglutinating activity of oregano and thyme extracts. This suggests the presence of lectins or lectin-like compounds in these plant extracts, as lectins are well known for their ability to cause erythrocyte agglutination. The observation that the thyme extract exhibited more pronounced agglutination compared to the oregano extract raises intriguing questions about the potential role of lectins in these extracts. This difference in agglutination activity could be attributed to variations in the lectin concentrations or lectin types between the two plant species [27].

Lectins have been studied extensively for their immunomodulatory potential and anti-inflammatory, antiviral, and anticancer effects [28], and their presence in oregano and thyme extracts suggests it is possible that these plant compounds can influence the immune system. Future research could delve into the specific mechanisms by which these lectins may impact immune responses.

### 2.4. Hemolytic Tests

#### 2.4.1. Evolution of Hemolytic Effect of Plant Extracts

This study involved a biological investigation of in vitro cytotoxicity based on the hemolysis of rat red blood cells during subjection to various concentrations of plant extracts from *O. grosii* and *T. pallidus*. The test of the hemolytic effect of the extracts was conducted in vitro using a red blood cell suspension from rat blood, which was incubated in a phosphate-buffered saline (PBS) solution at pH 7.4.

**Figure 7:**
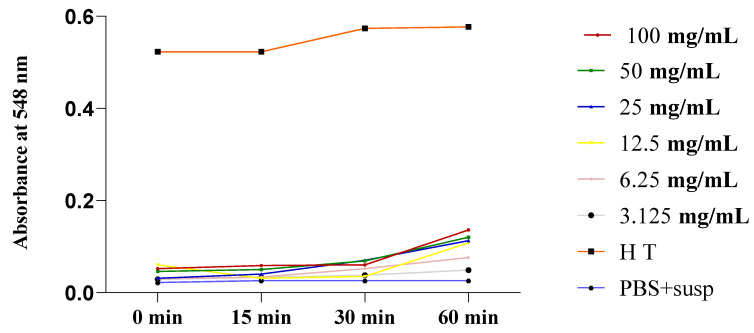
Absorbance evolution over time in tubes containing red blood cell suspension in the presence of various concentrations of total extract from *O. grosii* and HT, incubated at 37 °C.

**Figure 8:**
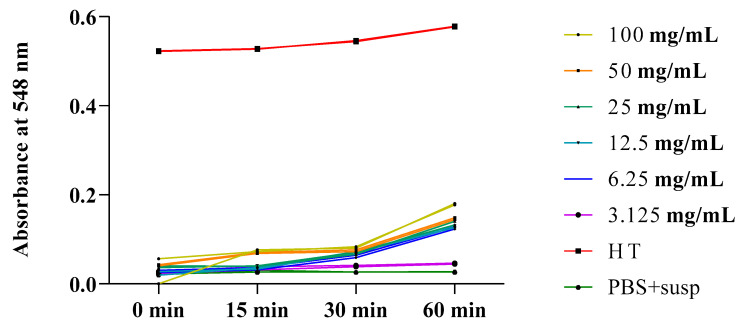
Absorbance evolution over time in tubes containing red blood cell suspension in the presence of various concentrations of total extract from *T. pallidus* incubated at 37 °C.

According to the obtained results, we observed increases in absorbance (hemolysis rates) during the 60 min incubation of isolated erythrocytes in PBS (pH 7.4) in the presence of different concentrations of the plant extracts. Figure 7 and Figure 8 depict the evolution of the hemolytic effect over time, as measured by absorbance, over 60 min. This took place in a PBS buffer medium (pH 7.4) containing a red blood cell suspension; the medium was incubated at 37 °C and exposed to various concentrations (3.125, 6.25, 12.5, 25, 50, and 100 mg/mL) of the total extracts from *O. grosii* and *T. pallidus*. These results were compared to those for a negative control (a tube containing only PBS and the red blood cell suspension) and a total hemolysis control (HT) triggered by distilled water.

Based on these results, we observed an increase in absorbance over time (0, 15, 30, and 60 min) as the concentration of *O. grossi* extract increased (Figure 7). This indicates an increase in the hemolysis rate with higher extract concentrations. But as we can see, not all exceeded an absorbance of 0.2, even for the highest concentration (100 mg/mL).

Similarly, for *T. pallidus*, we observed that absorbances also increased with varying concentrations. According to the obtained results (Figure 8), we recorded increases in absorbance (hemolysis rate) during the 60 min incubation of isolated erythrocytes in PBS (pH 7.4) with different concentrations of thyme total extract. We noted that absorbances also increased with varying concentrations, but they did not exceed a value of 0.2 for all concentrations except 100 mg/mL.

The results we obtained in this test, as well as a comparison of the absorbances of our extracts from oregano and thyme with the absorbance for total hemolysis, allow us to conclude that our samples do not cause red blood cell hemolysis. They are not toxic, a finding that is consistent with previous research [29,30]. Therefore, it is essential to exercise caution when consuming potentially toxic substances such as plants to avoid harming one’s health.

#### 2.4.2. Hemolysis Rate Assessment

**Figure 9:**
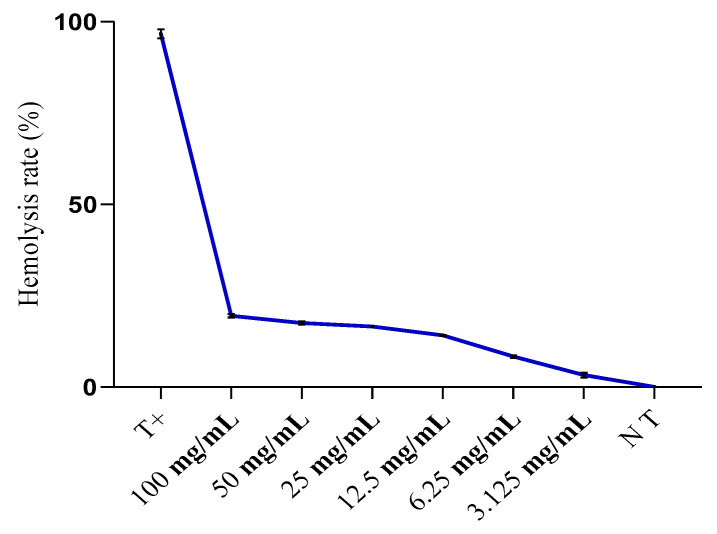
Hemolysis rate (%) evolution of different concentrations of total extract from *O. grosii* extract after 60 min of incubation compared to total hemolysis. NT: negative control; T+: positive control.

Figure 9 displays the hemolysis rates, expressed as percentages, following a 60 min incubation at 37 °C in a PBS buffer (pH 7.4). These incubations involved erythrocyte suspensions and various concentrations of oregano extract, with results compared to a control group consisting of an erythrocyte suspension induced by distilled water (total hemolysis). The hemolysis rates observed are relatively low, gradually increasing with increasing concentrations of the oregano extract. At the highest concentration tested (100 mg/mL), the hemolysis rate reached approximately 19.54%, as compared to the positive control (total hemolysis).

**Figure 10:**
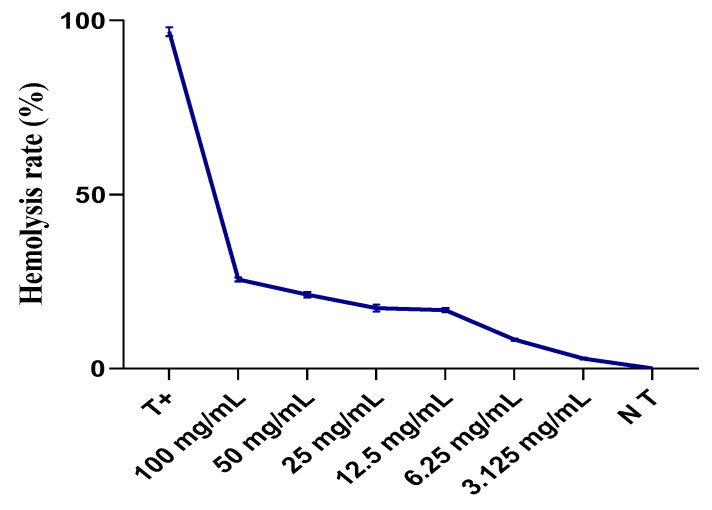
Evolution of hemolysis rate (%) of different concentrations of total extract from *T. pallidus* extract after 60 min of incubation compared to total hemolysis. NT: negative control; T+: positive control.

The outcomes from the evaluation of the hemolytic impact of the complete thyme extract (Figure 10) indicate that our extract demonstrates a minimal toxic effect on isolated erythrocytes. Specifically, at a concentration of 3.125 mg/mL, the hemolysis rate did not surpass 2.855 ± 0.361% when compared to total hemolysis. However, it did increase to 25.58 ± 0.82% at a concentration of 100 mg/mL.

The data indicate that the hemolysis rate for thyme at the highest concentration (100 mg/mL) was 25.58%, which is higher than the hemolysis rate for oregano at the same concentration, which was 19.54%. Consequently, the data suggest that thyme exhibits a higher level of toxicity than oregano at this concentration. It is important to note that even at high concentrations, the hemolysis rates remain below 30%, which can be considered a relatively low toxicity threshold for these extracts. As a result, they could serve as significant sources in therapeutic and pharmacological fields for alleviating various diseases.

### 2.5. Anti-Inflammatory Effect of O. grosii and T. pallidus Total Extracts

The table below provides information on the percentage inhibition of inflammation in mice following the administration of oregano extract at two different doses (400 and 800 mg/kg), thyme extract at two different doses (400 and 800 mg/kg), and indomethacin at a dose of 10 mg/kg (Table 5).

Oregano, administered at 400 and 800 mg/kg doses, showed a dose-dependent inhibition of inflammation over time. At both doses, oregano extract displayed significant anti-inflammatory activity, with higher doses resulting in more pronounced inhibition. The percentage of inhibition reached 94.23% at 6 h with the 800 mg/kg dose. This suggests that oregano extract effectively reduces inflammation, and its efficacy is indeed dose-dependent. Similar to oregano, thyme extract exhibited a dose-dependent inhibition of inflammation over time. At both doses (400 and 800 mg/kg), thyme extract displayed significant anti-inflammatory effects. The higher dose of 800 mg/kg resulted in higher inhibition percentages, with up to 86.53% inhibition at 6 h. This demonstrates the effectiveness of thyme extract in reducing inflammation, and its efficacy is also dose-dependent. Our findings align with those of previous research indicating the potential anti-inflammatory benefits of oregano and thyme extracts [31,32,33].

In comparison to the positive control, indomethacin, our study suggests that oregano and thyme extracts can be equally effective as anti-inflammatory agents. While indomethacin demonstrated consistent inhibition across all time points, both herbal extracts were competitive, particularly at the 800 mg/kg dose. These results are encouraging as they indicate that herbal extracts can be explored as natural alternatives to conventional anti-inflammatory drugs.

### 2.6. Analgesic Effect of O. grosii and T. pallidus Total Extracts

The study aimed to assess the pain-relieving effects of tramadol, oregano extract (at 400 and 800 mg/kg doses), and thyme extract (at 400 and 800 mg/kg doses) compared to a control group. Pain was measured according to induced abdominal contortions, with inhibition percentages indicating effectiveness.

Tramadol induced moderate pain relief at 10 mg/kg (47.69% inhibition). In contrast, both oregano and thyme extracts exhibited substantial and dose-dependent pain relief. Oregano extract achieved 72.56% (400 mg/kg) and 97.83% (800 mg/kg) inhibition, while thyme extract showed 69.48% (400 mg/kg) and 90.54% (800 mg/kg) inhibition (Table 6).

Compared to tramadol, both oregano and thyme extracts demonstrated the ability to induce significant pain relief, especially at higher doses. Oregano extract at 800 mg/kg induced an impressive 97.83% inhibition, surpassing that of tramadol. Similarly, thyme extract at 800 mg/kg achieved a significant 90.54% inhibition; these results corroborate earlier studies on oregano and thyme extracts, reaffirming their pronounced efficacy in alleviating pain [34,35,36,37,38].

Furthermore, it is essential to note that numerous studies have investigated the toxicity of oregano and thyme, consistently demonstrating their safety profiles and even highlighting their hepatoprotective effects [39,40,41,42,43]. These collective findings not only underscore the potential of oregano and thyme extracts as highly effective agents for pain relief, particularly at higher doses, but also strengthen their position as promising and safe alternatives or complementary options to traditional analgesic medications.

## 3. Materials and Methods

### 3.1. Plant Material

In June 2018, fresh leaves of *Origanum grosii* (*O. grosii)* and *Thymus pallidus* (*T. pallidus)* were systematically collected from the Ribat El Kheir region, located approximately 75 km from Fez, Morocco (33°49′ north, 4°25′ west). The identification of the plant specimens was conducted by an expert botanist associated with the Department of Biology, Sidi Mohamed Ben Abdellah University Fez, Morocco. Each plant specimen was assigned a voucher number: A51/08/06/2018/SE for *O. grosii* and A52/08/06/2018/SE for *T. pallidus*. After collection, the leaves were air-dried under suitable shade conditions to preserve their phytochemical compositions. Subsequently, the dried leaf samples were stored in a dry and cool environment at a temperature of 5 °C until they were ready for further analysis or experimentation.

The extraction of compounds from the air-dried leaves of *Origanum grosii* (*O. grosii*) and *Thymus pallidus* (*T. pallidus*) was carried out using a sonication-assisted method with a water–ethanol solvent mixture (70:30) at a temperature of 25 °C for a duration of 45 min. Subsequently, the resulting extracts were subjected to vacuum filtration. To concentrate the extracts, a rotary evaporator was employed, maintaining a temperature below 40 °C to prevent thermal degradation of the compounds.

### 3.2. Animal Material

A total of 60 Swiss Webster mice, both male and female, with a weight range of 30 to 35 g, were utilized as the experimental models in our investigations focused on assessing analgesic and anti-inflammatory properties. The animals were sourced from the animal facility in the Department of Biology, Faculty of Sciences Dhar El Mahraz, Sidi Mohamed Ben Abdellah Fes University, Morocco. Following their initial weight measurements, the mice were placed individually in polypropylene plastic cages within a controlled environment, featuring a 12 h light and 12 h dark cycle. They were provided with continuous access to clean, fresh water and were offered a nutritionally balanced diet.

For each experimental trial, the mice were categorized into six distinct groups, as delineated below: Group 1 was designated as the negative control, Group 2 was designated as the positive control, Group 3 received a 400 mg/kg dosage of oregano extract, Group 4 received 800 mg/kg of oregano extract, Group 5 was administered 400 mg/kg of thyme extract, and Group 6 was administered 800 mg/kg of thyme extract.

The methods used in this study comply with the international guidelines governing the use of laboratory animals under reference 04/2019/LBEAS. Throughout these experiments, all procedures involving the mice, as well as the associated protocols, were meticulously conducted in strict adherence to ethical standards and regulatory guidelines, ensuring the welfare and well-being of the animals were prioritized [44].

### 3.3. Phytochemical Analysis

This study’s methodology was based on a previous comparative study by Manal Zefzoufi et al. [45]. Notably, an Ultra-High-Performance Liquid Chromatography (HPLC) with Diode Array Detection and Electrospray Ionization Mass Spectrometry (UHPLC-DAD-ESI/MS) system called the Ultimate 3000 produced by Dionex, Sunnyvale, CA, USA, was used to perform the analysis. This system consisted of a quaternary pump, an autosampler (WPS 3000 TSL), and a column oven (TCC 3000). A Kinetex C18 reversed-phase column (250 mm; 4.6 mm; 2.6 μm particles) was used for chromatographic separation. The protocol involved a gradient elution with two solvents: a formic acid aqueous solution (solvent A) and methanol (solvent B). The gradient program was as follows: from 0 to 6 min, a linear gradient from 15% to 25% B was applied; from 6 to 12 min, holding at 25% B was conducted; from 12 to 15 min, an increase from 25% to 37% B was implemented; from 15 to 20 min, an increase from 37% to 95% B was induced; from 20 to 25 min, the initial conditions were restored at 15% B. The flow rate in the mobile phase was maintained at 1 mL/min. UV-Vis spectral measurements were collected in the 200–400 nm range, and chromatographic profiles were recorded at 280 nm. The mass spectrometer used was a triple quadrupole (TSQ) Endura (Thermo Fisher Scientific, San Jose, CA, USA) capable of heated-electrospray ionization in negative mode (H-ESI). Nitrogen (99.98%) was used as the sheath gas, ion sweep gas, and auxiliary gas at flow rates of 65, 0, and 40 arbitrary units, respectively. The vaporizer temperature for H-ESI and the temperature of the ion transfer tube were both adjusted to 350 °C. The electrospray voltage was set to −2.5 kV. We primarily utilized the full-scan MS acquisition mode within the Q1 mass range of *m/z* 50–1000, with a mass resolution of 0.7 *m/z* full-width half-maximum (FWHM) and a scan time of 0.5 s, for characterization and assessment. The method of structure assignment was based on comparing mass data with two libraries: an external library, specifically the NIST MD Search 2.3 library (MS library: MSMS library), and an internal library composed of a multi-stock solution of standards.

### 3.4. Antioxidant Activity

#### 3.4.1. Total Antioxidant Capacity (TAC)

In the TAC test, a 25 µL sample of the extract was combined with a reagent solution composed of 0.6 M of sulfuric acid (H_2_SO_4_), 28 mM of sodium phosphate (Na_2_HPO_4_), and 4 mM of ammonium molybdate ((NH_4_)_2_MoO_4_). This mixture underwent incubation at 95 °C for 90 min. Subsequently, the absorbance was measured at 695 nm using a spectrophotometer. To determine the total antioxidant capacity, the extract’s absorbance was compared to a standard curve of ascorbic acid. The results were quantified in micrograms of ascorbic acid equivalent per gram of extract (mg Eq BHT/g DW), offering an evaluation of the extract’s antioxidant potential based on the behavior of ascorbic acid under similar conditions [46].

#### 3.4.2. Beta-Carotene Bleaching Test

The β-carotene bleach inhibition test, adapted from Ozsoy et al. (2008) [47], with modifications, was used to assess the ability to inhibit β-carotene bleaching using a β-carotene/linoleic acid model. Linoleic acid (10 μL) and Tween 80 (100 μL) were mixed in a vial, and then a β-carotene solution (1 mL) obtained by dissolving 2 mg of β-carotene powder in 10 mL of chloroform was added. After removing the chloroform via rotary evaporation, 25 mL of 30% hydrogen peroxide was added to the residue, creating a stable emulsion.

Next, 2.5 mL of the emulsion was mixed with 100 μL of diluted extract or ascorbic acid (reference antioxidant) at a concentration of 35 mg/mL. A methanol control was prepared in the same way. Absorbance was measured at 470 nm against an empty test at t0. Tubes were placed in a water bath at 50 °C for various intervals (0 min, 60 min, 90 min, 120 min, 150 min, and 180 min) until β-carotene visually disappeared. The bleaching rate was calculated by comparing the initial absorbance with the final absorbance. Antioxidant activity was expressed as inhibition of β-carotene bleaching:

AA = % inhibition = [1 − (As0 − As)/Ac0 − Ac)] × 100(1)

where As0 and As represent the initial absorbance for the test and control; Ac0 and Ac represent the absorbance after 180 min.

#### 3.4.3. DPPH Scavenging Ability

The antioxidant potential of the extracts was assessed according to their ability to scavenge the stable free radical 1,1-diphenyl-2-picrylhydrazyl (DPPH) using a method adapted from Wu W et al. (2007) [48]. In essence, varying concentrations of the extract or a standard antioxidant were added (0.1 mL) to a mixture of DPPH methanolic solution (0.004%, 0.75 mL) and methanol (0.65 mL). This mixture was vigorously shaken and then left in the dark at room temperature. After 30 min of incubation, absorbance was measured at 517 nm. For comparison, the synthetic antioxidant BHT was used as a positive control. Percent inhibition was calculated using the following equation:

DPPH trapping capacity (%) = [(A0 − A1)/A0] × 100(2)

where A0 represents the absorbance of the control after 30 min, and A1 denotes the absorbance of the sample after the amount of same time.

### 3.5. Hemagglutination and Hemolytic Tests

#### 3.5.1. Preparation of Total Extract

Water-soluble substances were extracted from *O. grosii* and *T. pallidus* powders using phosphate-buffered saline (PBS). A total of 20 g of dried leaf powder was added to 200 mL of PBS buffer. The mixture was stirred for 2 h. After centrifugation at 3000 rpm for 15 min, the supernatant was collected and stored at 4 °C. This supernatant constitutes the total extract used for the biological tests, namely, the hemagglutination test and the hemolysis test.

#### 3.5.2. Preparation of Erythrocytes

Rat blood was collected in a heparinized test tube to prevent coagulation. The blood was then centrifuged at 5000 rpm for 3 min. The supernatant was discarded, and a physiological solution (NaCl 0.9%) was added to the packed erythrocytes at the bottom of the tube. This washing process was repeated four times under the same conditions. After the fourth wash, the red blood cells were diluted with 0.9% NaCl to obtain a 3% erythrocyte solution. To perform this, 1.5 mL of conditioned erythrocytes was mixed with 48.5 mL of 0.9% NaCl solution.

#### 3.5.3. Hemagglutination Test

The hemagglutination test is performed to detect the presence of lectins in extracts. This test is based on the observation of agglutination and precipitation of erythrocytes in the presence of lectins. The test is performed on 96-well round-bottom plates. Amounts of 50 μL of 0.9% NaCl, 25 μL of test extract, and 25 μL of 3% erythrocytes are added to each well. The plate is covered and incubated at 37 °C for 30 min before the results are read. Two controls are used: A positive control (Concanavalin A) and a negative control prepared by mixing 75 μL of 0.9% NaCl with 25 μL of 3% erythrocyte suspension [49].

#### 3.5.4. Hemagglutination Limit

This test aims to determine the minimum concentration of extract that causes erythrocytes to agglutinate. The same protocol as above is used, but this time, a range of dilutions for each extract are employed, spanning from 1/5 to 1/2000.

#### 3.5.5. Hemolytic Activity

We studied the in vitro hemolytic effects of *O. grosii* and *T. pallidus* extracts using rat erythrocytes because of their simplicity with regard to assessing cytotoxicity. The evaluation followed a method modified from [50,51]. Blood was collected and centrifuged, and the erythrocytes were suspended in PBS. Varying concentrations of extracts were mixed with the erythrocyte suspensions and incubated. Absorbance was measured over time, and hemolysis rates were calculated as a percentage of total hemolysis after one hour, using concentrations such as 3.125 mg/mL, 6.25 mg/mL, 12.5 mg/mL, 25 mg/mL, 50 mg/mL, and 100 mg/mL.

### 3.6. Anti-Inflammatory Effect of O. grosii and T. pallidus Total Extracts

This activity was conducted following the method described by [52], with slight modifications. After oral administration of the different doses of the two plants to the mice with volumes between 0.3 mL and 0.35 mL (depending on the weight of the mice), the paw volume was measured initially before the injection of 0.5% carrageenan into the left paws of the mice. The paw volume was then periodically measured at 3, 4, 5, and 6 h after the injection, using a digital caliper, and the results were recorded in millimeters (mm). The inhibitory activity was calculated using the following equation:

(3) $$PI=\left(1-\left(\frac{a-x}{b-y}\right)\right)\times 100$$

The following is a description of the terms used in the equation above:

*PI* is percent inhibition;

*a* is the average paw volume of a treated mouse after carrageenan injection;

*x* is the average paw volume of a treated mouse before the injection;

*b* is the average paw volume of the control mouse after carrageenan injection;

*y* is the average paw volume of the control mouse before the injection.

### 3.7. Analgesic Effect of O. grosii and T. pallidus Total Extracts

To investigate the analgesic properties of our extracts, we implemented a similar experimental setup as previously described, utilizing mice as our in vivo study subjects. Oral administration was carried out on both male and female mice, randomly allocated into 6 groups, each consisting of 5 mice. Approximately one hour later, an intraperitoneal injection of a 1% acetic acid solution was administered at a rate of 10 mL/kg. Subsequently, writhing movements were meticulously recorded during a 30 min observation period, following established protocols [53,54].

### 3.8. Statistical Analysis

The data are displayed as averages along with their corresponding standard deviations (SD), which were computed from three independent repetitions. We utilized GraphPad Prism (version 8.0.1) to conduct statistical evaluations. The normality of the data distribution was assessed using the Shapiro–Wilk test, while Levene’s test was employed to examine the equality of variances. To ascertain significant differences among the means, we performed analysis of variance (one-way ANOVA), followed by Tukey’s post hoc test for multiple comparisons. The significance level was set at *p* ≤ 0.05.

## 4. Conclusions

In conclusion, the phytochemical analysis and array of biological assays conducted have unveiled the multifaceted potential of these extracts. The identified compounds, Licoflavone C and hesperidin, provide valuable insights into the compositions of our extracts. *O.grosii* displayed notable antioxidant activity, with an IC50 of 0.085 mg/mL in the DPPH assay, outperforming *T. pallidus*. Additionally, *O. grosii* exhibited significant analgesic properties, with a remarkable 97.83% spasm inhibition, compared to *T. pallidus* at 90%. Both extracts showed limited hemolytic activity, not exceeding 30% at a concentration of 100 mg/mL, indicating their potential safety.

These findings highlight the potential applications of these extracts in pharmacological research. Their diverse range of bioactivities, including antioxidant and analgesic effects, suggests promising therapeutic applications. Further investigations are warranted to fully explore their potential and facilitate the development of novel treatments for various health conditions.

## Figures and Tables

**Table 1:** Phytochemical screening of *O. grosii* and *T. pallidus* total extracts.

N°	RT	UV	*m*/*z* [M-H]^−^	Identified Compounds	% Area		% Match
					*O. grosii*	*T. pallidus*	
1	5.03	211, 267	353.9	4-Caffeoylquinic acid	-	2.62	59%
2	11.66	222, 320	367	4-*O*-Feruloylquinic acid	1.96	10.08	55%
3	15.18	296, 340	174.9	Methoxycoumarin	-	2.5	52%
4	18.01	211	755	Kaempferol-3-*O*-rutinoside-7-*O*-glucoside	-	6.04	69%
5	18.72	227, 274, 354	609.17	hesperidin	16.9	43.57	77%
6	21.22	257, 357	609	Rutin	20.93	3.38	82%
7	22.9	215, 285	593	kaempferol-rutinoside	-	4.8	65%
8	26.79	278, 360	739	unknown	26.51	6.59	66%
9	28.2	260	337.4	Licoflavone C	30.96	11.22	62%

**Table 2:** Antioxidant activity: oregano and thyme extracts vs. positive controls (DPPH and FRAP).

	*O. grosii*	*T. pallidus*	BHT	Quercetin
IC_50_ (mg/mL)	0.085 ± 0.004 ^a^	0.146 ± 0.02 ^b^	0.11 ± 0.001 ^c^	-
EC_50_ (mg/mL)	0.167 ± 0.02 ^a^	0.185 ± 0.04 ^a^	-	0.03 ± 0.004 ^b^

Data are presented as mean values ± SD of three measurements. Each line in the table with the same letter (a, b, or c) does not represent a significant difference (*p* < 0.05).

**Table 3:** Agglutination of rat red blood cells by crude aqueous extract.

Plant	Agglutination Test
*O. grosii*	++
*T. pallidus*	+++

+++: Very strong agglutination; ++: strong agglutination.

**Table 4:** Hemagglutinating activity of *O. grosii* and *T. pallidus* extracts.

	Dilution								
	SM	1/5	1/10	1/20	1/50	1/100	1/200	1/400	1/1000
*O. grosii*	+++	++	++	+	-	-	-	-	-
*T. pallidus*	+++	+++	++	++	+	-	-	-	-

+++: Very strong agglutination, ++: strong agglutination, +: weak agglutination, and -: absence of agglutination. SM: initial solution.

**Table 5:** Percentage inhibition of inflammation in mice for *O. grosii* extract (400 and 800 mg/kg), *T. pallidus* extract (400 and 800 mg/kg), and Indomethacin (10 mg) (PC: positive control; SD: standard deviation).

Average Diameter ± SD (cm), % Inhibition of Inflammation ± SD							
	Dose (mg/kg)		0 h	3 h	4 h	5 h	6 h
Control		Di (cm)	1.33 ± 0.1	1.87 ± 0.2 *	1.83 ± 0.2 *	1.77 ± 0.2 *	1.73 ± 0.1 *
Indomethacin	10	Di (cm)	1.30 ± 0.06	1.5 ± 0.06 ^ns^	1.4 ± 0.06 ^ns^	1.03 ± 0.08 **	0.6 ± 0.1 ****
		In (%)	0.0 ± 0.0	19.64 ^a^	23.63 ^a^	41.5 ^a^	65.38 ^a^
*O. grosii*	400	Di (cm)	1.36 ± 0.1	1.2 ± 0.2 **	0.86 ± 0.1 **	0.4 ± 0.2 ****	0.12 ± 0.1 ****
		In (%)	0.0 ± 0.0	35.71 ^b^	52.72 ^b^	73.58 ^a^	92.3 ^b^
	800	Di (cm)	1.5 ± 0.1	1.06 ± 0.1 **	0.9 ± 0.1 **	0.36 ± 0.1 ****	0.1 ± 0.1 ****
		In (%)	0.0 ± 0.0	42.85 ^b^	50.9 ^b^	79.24 ^a^	94.23 ^b^
*T. pallidus*	400	Di (cm)	1.3 ± 0.1	1.1 ± 0.04 **	0.86 ± 0.07 **	0.63 ± 0.07 ***	0.36 ± 0.03 ****
		In (%)	0.0 ± 0.0	41.07 ^b^	52.72 ^b^	64.15 ^a^	78.84 ^b^
	800	Di (cm)	1.43 ± 0.1	1.16 ± 0.2 **	0.83 ± 0.2 ***	0.5 ± 0.1 ****	0.23 ± 0.1 ****
		In (%)	0.0 ± 0.0	37.5 ^b^	54.54 ^b^	71.69 ^a^	86.53 ^b^

Statistical analysis was carried out between the negative control (NaCl, 09%) and the other samples (Indomethacin, *O. grosii*, and *T. pallidus*), resulting in each column bearing the same number of stars (*, **, ***, ****; *p* < 0.05), meaning that there was no significant difference in diameter (Di) when compared with the negative control. And a second statistical analysis was performed on the positive control (indomethacin) and *O. grosii* and *T. pallidus*; here, the existence of colonies bearing the same letter (a or b) does not indicate a significant difference in the percentage of inhibition (In) when compared with the positive control. ns: not significant.

**Table 6:** Results regarding analgesic activity, percentage of analgesic inhibition (% ± SD), the average number of spasms, percentage of analgesic inhibition (number ± SD), and Tramadol 10 mg (PC).

Treatment	Dose (mg/kg)	Number of Contortions	Inhibition (%)
Negative control	NaCl (0.9%)	75.78 ± 5.2 ^a^	-
Tramadol	10	39.64 ± 3.04 ^b^	47.69 ^a^
*O. grosii*	400	20.79 ± 3.84 ^c^	72.56 ^b^
	800	1.64 ± 0.64 ^d^	97.83 ^c^
*T. pallidus*	400	23.13 ± 3.28 ^c^	69.48 ^b^
	800	7.17 ± 1 ^d^	90.54 ^c^

Data are presented as mean values ± SD of three measurements. Statistical analysis was performed between the negative control (NaCl) and the other samples (Tramadol, *O. grossi* and *T. pallidus*) and between the positive control (Tramadol) and the two extracts (*O. grossi* and *T. pallidus*). Values bearing the same letter (a, b, c, or d) in the same column are significantly different (*p* < 0.05).

## Data Availability

Data are contained within the article.
